# Single-crown restorations in premolar–molar regions: short (≤ 6.5) vs longer implants: retrospective cohort study

**DOI:** 10.1186/s40729-022-00438-y

**Published:** 2022-10-04

**Authors:** Eduardo Anitua, Mohammad Hamdan Alkhraisat, Asier Eguia

**Affiliations:** 1 Clínica Eduardo Anitua, Jose Maria Cagigal Kalea, 19, 01007, Vitoria-Gazteiz, Álava Spain; 2 BTI-Biotechnology Institute, Vitoria-Gazteiz, Álava Spain; 3 University of the Basque Country UPV/EHU, Leioa, Vizcaya Spain

**Keywords:** Dental implants, Short implants, Marginal bone loss, Crown-to-implant ratio

## Abstract

**Purpose:**

To compare the survival, changes in marginal bone level and prosthetic complications rate of short (≤ 6.5 mm) and longer implants (≥ 7.5) supporting a single-crown restoration in the maxillary/mandibular premolar or molar region.

**Methods:**

This cohort study was conducted following the STROBE statement recommendations for observational studies. Clinical outcomes of 88 short implants in 78 patients and 88 long implants in 88 patients were examined. All the implants had been placed by the same surgeon and restored following the same prosthetic concept; using a transepithelial abutment (intermediate abutment) and a screw retained restoration.

**Results:**

All the implants were in function after the follow-up period since insertion (median: 31 months; range 11 to 84 for SiG vs median: 35 months; range: 6–117 for CG; *p* = 0.139). No statistical differences (*p* = 0.342) were observed related to prosthetic complications (screw loosening 2/88 vs 5/88 CG, ceramic chipping 1/88 vs 0/88, temporary crown resin chipping 1/88 vs 0/88 for SiG and CG, respectively) or related to marginal bone level (Mesial or Distal MBL ≥ 2 mm in 1/88 implants for SiG vs 3/88 for CG; p = 0.312).

**Conclusions:**

Within the limitations of this study, no survival differences have been observed between short implants and longer implants in single-crown restorations in posterior maxilla/mandible.

## Introduction

Tooth loss leads to changes in the integrity of the alveolar bone and soft tissues [1, 2]. The healing of extraction sockets leads to histological and dimensional changes in the remaining alveolar ridge [1–4]. Progressive atrophy of the alveolar process begins at this moment both horizontally and vertically. Resorption of the alveolar ridge occurs due to a combination of different factors, such as loss of the periodontal ligament (and lack of the vascularization of the bundle bone), loss of function (and the stimulus on the bone), fractures in the alveolar wall during extraction or the subsequent occurrence of infectious processes [1–4].

The physiological post-extraction resorption (RRR, Residual Ridge Reduction) [5] can hinder the implant rehabilitation of missing teeth. It not only affects the available volume, but also the density of the remaining bone [5]. These changes could hamper the subsequent placement of an implant in an optimal position or affect the esthetic results. [6]

Clinicians are often faced with the challenge of treating patients with severe vertical bone atrophy. Different treatment options allow the use of standard implants in the posterior region where the nerve canal or the maxillary sinus limit the residual bone height, such as guided bone regeneration (GBR), maxillary sinus grafting, inlay or onlay bone graft, distraction osteogenesis, nerve lateralization or others [7, 8]. These techniques require greater knowledge and surgical skills on the part of the professional and potentially increase the complication rate [9–12]. Recent advances in implant design and sizing (short, extra-short implants) have provided new solutions and alternatives or have allowed optimization of existing ones [13–15]. In addition to facilitate the procedures, the use of short implants reduces the risk of reaching anatomical structures at the time of drilling, minimizes the number of surgeries, reduces the time and cost of treatment, and saves the need for bone augmentation. [7, 16–18]

In cases of vertical atrophy, the prosthetic space is larger thus increasing the crown-to-implant ratio. Former recommendations about the ideal proportions seem outdated in sight of the diverse clinical and biomechanical studies demonstrating the safety and good clinical performance of short and extra-short implants [19, 20]. Crown-to-implant ratios ranging from 0.9 to 2.2 did not influence the occurrence of technical or biological complications [21]. Indeed, it has been claimed that within the range of 0.6 to 2.36, the higher the crown-to-implant ratio, the less the peri-implant marginal bone loss (MBL) [20]. As the length of the implant is reduced, it has been suggested to increase the diameter to enhance the bone–implant contact and optimize the distribution of stress in the bone, particularly in cases of low bone density. [22]

Short implants are not limited to cases of limited available bone. Currently, short implants can be preferred to maintain as much pristine bone as possible, even when standard implants could be housed. [23, 24]

Some authors reported that short implants could have lower survival rates than standard implants [25, 26] but recent systematic reviews have shown that short implants had a better or equal performance compared with standard [27–29] and did not seem to have a significant influence on marginal bone loss [30]. Several systematic reviews and meta-analysis have been conducted to clarify the controversies on the clinical performance of short implants [25, 31–34] but their results should be individually interpreted with caution to assess the eventual presence of uncontrolled confounding factors in the included studies [31] (as studies including both splinted and non-splinted restorations, implants placed in both grafted or pristine bone, different implant designs and surfaces or different types of restorative design).

There is also another controversy regarding the classification of short implants that could result in a misinterpretation of the results. While some authors considered short implants those under 10 mm [35, 36], others considered a length under 8 mm [37] and more recently, others under 6.5 mm [38] or 6 mm. [39, 40]

Recent evidence from clinical trials has shown the need for more studies and longer periods of follow-up before the recommendation of short implants to support single-crown restorations [19, 40]. The objective of this study has been the comparison between short implants and longer implants in terms of implant survival, marginal bone remodeling and prosthetic complications of single-crown restorations.

## Materials and methods

### Study design

The present unicentric observational retrospective study was conducted following the STROBE statement recommendations for observational studies and in compliance to the principles of the Declaration of Helsinki on clinical research involving human subjects. Before starting, the permission of the ethics committee was obtained from the Basque drug research committee.

### Sample size estimation

A study of a continuous response variable of matched pairs of study subjects was planned. The sample size was estimated based on previous bone loss data at 12-month follow-up indicating that the difference in response of matched pairs was normally distributed with a standard deviation of 0.4320 [41]. If the true difference in marginal bone loss at 12 months of matched pairs was 0.13, 88 pairs of implants should be necessary to reject the null hypothesis that this difference in response is zero with a probability (power) of 0.8. The probability of type I error associated with this test of the null hypothesis was 0.05.

### Patients

Data were retrospectively collected from 88 short (≤ 6.5 mm; 78 patients) and 88 longer implants (≥ 7.5 mm; 88 patients) randomly selected from a cohort of 16.780 implants placed from 2012 to 2019 at the same center (Eduardo Anitua Clinic, Vitoria, Spain). After sample size estimation, this cohort was divided in two groups (short implant group; SIG and control group; CG) and simple random sampling was conducted using the SPSS software, (SPSS for Windows, Version 15.0. Chicago, SPSS Inc) to select 88 implants form each group.

The inclusion criteria were:

- Implants placed both in maxilla or mandible
- Implants supporting a single-crown screw-retained, restored using a transepithelial (intermediate abutment).
- Patients over 18 years

To address sources of bias, all patients included in this study had been previously treated by the same team, using the same implant system (UnicCa®, BTI Biotechnology, Vitoria, Spain) and the same surgical and prosthetic protocols. All the treatments were performed following the usual clinical practice of the participating center for the insertion and subsequent loading of short and standard implants in the mandible and/or maxilla.

### Data collection methods

The outcomes measured were survival (presence of the implant at the last visit), MBL and prosthetic (technical) complications. The bone level assessment was performed vertically measuring the distance from the bone crest to the first bone-implant contact both mesially and distally. Measurements to estimate MBL were performed at loading time and at the time of the last available radiograph using the Sidexis software (Dentsply Sirona; York, US) and the length of the implant was used as calibrator. Among the technical complications screw loosening/break and ceramic/resin chipping were considered.

Other clinically relevant variables recorded were implant diameter, location, insertion torque, bone type, sex and age, residual bone height, type of antagonist teeth and the need for additional surgical techniques. Follow-up time was calculated since implant insertion (until last recall) and implant loading (until last recall).

The crown-to-implant ratio was determined by dividing the length of the crown together with the transepithelial (intermediate) abutment by the length of the implant. Residual bone height was measured from the bone ridge crest to the maxillary sinus/nerve canal at the implant position, using the radiography obtained previously to the surgery.

Information about smoking habit, alcohol intake, diabetes or hypertension was also retrieved from medical records. Bone type quality [42] was rated with the aid of computer software (BTI Scan, BTI Biotechnology, Vitoria, Spain).

### Statistical analysis

A statistical analysis was performed using specialized software (SPSS for Windows, Version 15.0. Chicago, SPSS Inc). Categorical variables were expressed in absolute and relative frequencies. Continuous variables were expressed as median and range. Before statistical analysis, the normal distribution of the continuous variables was evaluated using the Saphiro–Wilk normality test. Statistical differences between categorical variables were performed by the Chi-square test, and statistical differences between dichotomous and continuous categorical variables were performed with the Mann–Whitney test. The effect of the crown to implant ratio on the marginal bone loss was assessed by linear regression analysis. The statistical significance was set at *p* < 0.05.

## Results

The study included 176 dental implants placed in 166 patients that complied with the inclusion/exclusion criteria. The Short implant Group (SiG) was composed by 88 short implants (≤ 6.5 mm) placed in 78 patients (53 females; 35 males) and the Control Group (CG) was composed by 88 implants (≥ 7.5 mm) placed in 88 patients (52 females; 36 males). Further demographic data are presented in Table 1.

**Table 1:** Demographic data

	SiG	CG	*p*-value
Number of patients (*n* = 176)	78	88	NA
Number of implants (*n* = 186)	88	88	NA
Age (years; median (range)	56 (20 to 78)	53 (18 to 76)	0.159^a^
Sex (females (males)	52 (36)	53 (35)	0.878^b^
Smokers	7	8	0.787^b^

*CG* control group, *SiG* short implant group

^a^Mann–Whitney test

^b^Chi-square test

**Fig. 1:**
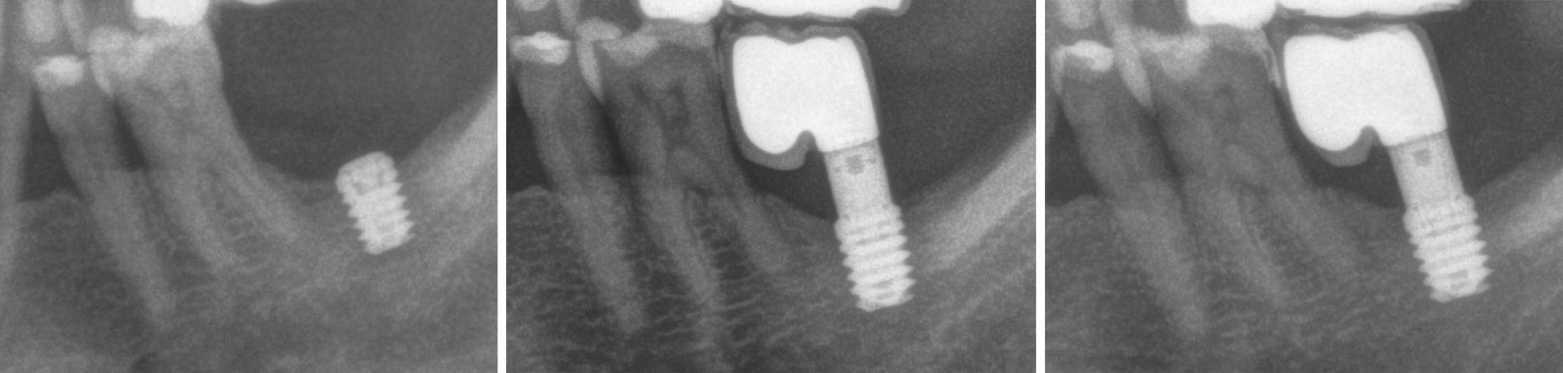
*Left*: 6.5 length implant placed in #3.7 position. *Center:* 2 years later; single screw-retained crown over 4 mm. straight transepithelial (intermediate abutment). Right: 6 years after implant placement. No marginal bone loss observed after 6-year follow-up

**Fig. 2:**
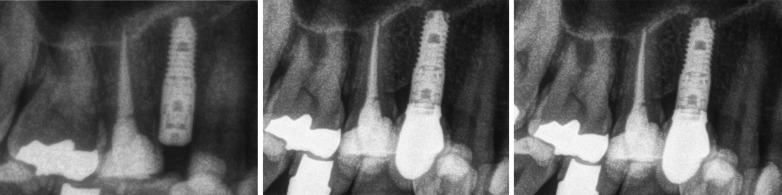
*Left*: 8.5 length implant placed in #1.4 position. *Center:* 2 years later; single screw-retained crown over 2 mm. Straight transepithelial (intermediate abutment). Right: 6 years after implant placement. No marginal bone changes after 6-year follow-up

**Fig. 3:**
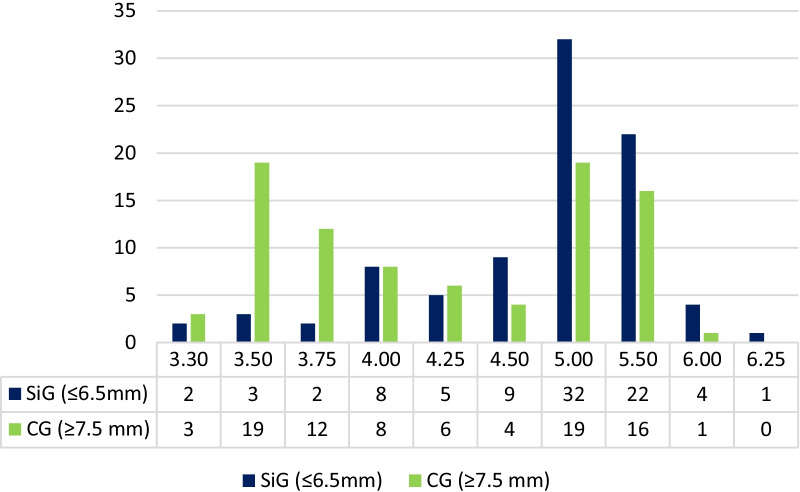
Implant diameter. SiG (short implant group), CG (control group)

From the SiG, 71 implants were 6.5 mm-length and 17, 5.5 mm-length. Attending to their location, 4 corresponded to Upper Premolars (UP), 3 to Lower Premolars (LP), 52 to Upper Molars (UM) and 29 to Lower Molars (LM). Figures 1,2. From the CG, 69 were 7.5-length, 17 8.5 mm-length and 2 were 10 mm-length, and corresponded to 23 UP, 11 LP, 20 UM and 34 LM. Diameter of the implants is presented in Fig. 3. The diameter of SiG implants was higher (*p* < 0.001).

**Fig. 4:**
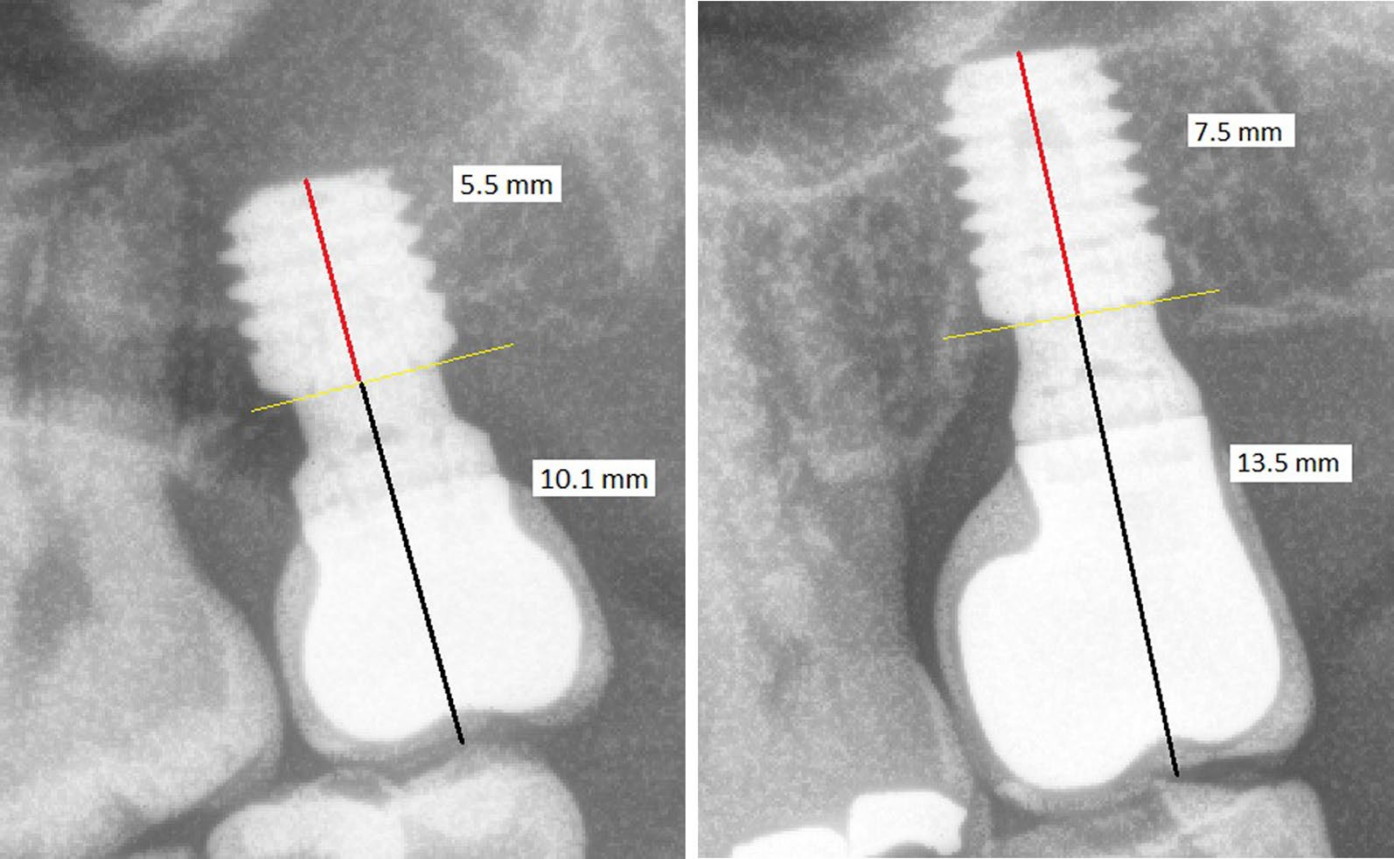
*Left*: 5.5 mm length implant placed in the #2.7 position. Screw-retained single-crown over a 3 mm transepithelial (intermediate abutment). Crown-to-implant ratio: 1.84. *Right*: 7.5 mm length implant placed in the same position (#2.7). Screw-retained single-crown over a 3 mm transepithelial (intermediate abutment). Crown-to-implant ratio: 1.80

Residual bone height was higher (p < 0.001) in CG (12.6 vs 7.7 mm) and crown-to-implant ratio was higher (*p* < 0.001) in SiG (1.7 vs 1.3 mm). Figure 4. Conversely, there were no statistical differences between both groups regarding insertion torque, bone type or antagonist type. Further information is available in Table 2.

**Table 2:** Surgical data, crown-to-implant ratio, and follow-up data

	Group		*p*-value
	Experimental	Control	
Residual bone height (mm; median (range))	7.7 (4.2 to 17.6)	12.6 (7.7 to 20.4)	0.000^a^
Insertion torque (Ncm; median (range))	42.5 (5.0 to 70.0)	35.0 (5.0 to 70.0)	0.188^a^
Bone type (number of implants)			
Type I	4	4	0.234^b^
Type II	44	49	
Type III	33	22	
Type IV	7	13	
Antagonist (number of implants)			
Tooth	63	66	0.609^b^
Implant	25	22	
Crown to implant ratio (median (range))	1.7 (1.1 to 2.5)	1.3 (0.9 to 2.1)	0.000^a^
Immediate loading (number of implants)	49	49	1.000^b^
Follow-up since insertion (months; median (range))	31 (11 to 84)	35 (6 to 117)	0.139^a^
Follow-up since loading (months; median (range))	27 (4 to 84)	31 (6 to 67)	0.249^a^
Follow-up of marginal bone level (months; median (range))	24 (3 to 92)	30 (1 to 96)	0.095^a^

^a^Mann–Whitney test

^b^Chi square test

At the time of implant placement, no differences could be observed attending to the number of implants placed equicrestal (− 0.5 to 0.5 mm), subcrestal > 0.5 mm or supracrestal < − 0.5 mm. in mesial, but a higher proportion of short implants (*p* ≤ 0.001) were placed equicrestal.

At the time of loading and last radiography, no differences in bone level were observed between both groups in mesial. CG implants were more subcrestal in distal at loading time than SiG implants (*p* ≤ 0.001). In addition, during the last radiography, a slight difference (*p* < 0.05) in distal bone level was observed in favor to CG implants. Moreover, the crown to implant ratio did not significantly affect the marginal bone loss (*p* = 0.781).

The follow-up of dental implants, since insertion had a median of 31 months (range 11 to 84) for SiG and 35 months for CG (range 6–117).

All the implants from both group were in function at the last recall (100% survival) and the number of prosthetic complication did not statistically differ (3 events for SiG vs. 6 for CG). Attending to the Health Scale for Dental Implants [43], Success rate for SIG group was 100% and 98.9% for CG. No statistical differences in Marginal Bone Loss (MBL) were observed either in mesial or distal between both groups after the follow-up period. There were no differences in the number of implant areas (mesial/distal) showing MBL ≥ 2 mm (SiG 1/166; 6/166 CG). Additional information about the clinical performance is available in Table 3.

**Table 3:** Results. Data related to implant survival, MBL (marginal bone loss) and prosthetic complications

		Group		*p* value
		Short Implants	Standard	
Technical complications (number of implants)	None	85	82	0.342^b^
	Screw loosening	2	5	
	Resin chipping	1	0	
	Ceramic chipping	0	1	
Mesial MB level (mm; median (range))	Loading	0.7 (− 0.7 to 2.5)	0.7 (− 0.4 to 2.3)	0.173^a^
	Last visit	0.8 (− 1.2 to 2.9)	0.6 (− 3.3 to 2.9)	0.262^a^
Distal MB level (mm; median (range))	Loading	0.3 (− 2.5 to 2.3)	0.6 (− 1.3 to 2.3)	0.001^a^
	Last visit	0.4 (− 2.1 to 2.1)	0.5 (− 3.8 to 2.4)	0.018^a^
Change in MB level (mm; median (range))	Mesial	0 (− 1.5 to 1.3)	0.1 (− 4.0 to 1.4)	0.322^a^
	Distal	0.1 (− 2.2 to 1.0)	0.1 (− 4.0 to 1.0)	0.758^a^
Change in MB level ≥ 2 mm	Mesial	0	1	0.316^b^
	Distal	1	3	0.312^b^
Change in MB level ≥ 3 mm	Mesial	0	1	0.316^b^
	Distal	0	1	0.316^b^
Survival (number of implants)		88	88	NA

Marginal bone (MB) level ((−): below the most coronal part of the implant shoulder; ( +): above the most coronal part of the implant shoulder)

^a^Mann–Whitney test

^b^Chi square test

## Discussion

The results of the present retrospective cohort study showed no differences in survival, changes in marginal bone levels or prosthetic complications between SiG and CG implants supporting a single-crown restoration over a transepithelial (intermediate abutment) in posterior maxilla or mandible. These results are in line with those of the systematic review and meta-analysis published by Tolentino da Rosa de Souza et al. [7] Short implants in posterior single crown had similar survival rates, low MBL and low prosthetic and surgical complications rate for a 1-year follow-up time. On the contrary Xu et al. [41] on their meta-analysis stated that the survival rate of short implants in the maxilla may be lower than that of long implants, while in the mandible both type of implants showed similar survival rate. Furthermore, short implants have been associated with lower MBL and biological complications but higher technical complications. It Worth mentioning that short and longer implant definition was different in these studies [7, 41]. Moreover, most of authors selected a unique length in the SiG and a unique length in the CG instead of defining a limit length between both groups. The type of included articles (only RCTs [41] or CCTs and RCTs [7]), the ratio of maxillary/mandibular implants, the proportion of implants placed with additional techniques, or the follow-up period could also explain the differences between both meta-analysis.

Systematic reviews comparing Short and Standard implants including both splinted and non-splinted implants [31, 44, 45] should be interpreted with caution as the biomechanical performance of splinted implants is substantially different from non-splinted ones. In multiple restorations splinted implants can disperse the stress on each single implant thereby reducing the implant overload and the possible incidence of mechanical complications [7, 46, 47]. In relation to the biomechanical performance of short implants supporting single crowns, similar outcomes have been reported when the implant is placed in the most distal position in the arch or between adjacent teeth or other implants. [48]

Many articles have been published comparing the performance of short implants in native bone vs longer implants along with sinus grafting in the maxilla. A recent umbrella review of meta-analysis from Vetromilla et al. [28] concluded that short implants showed fewer biological complication rates, reduced cost, and an overall similar satisfaction rate of the patients.

In the present study, the crown-to-implant ratio of all the implants in the CG where within the ranges that have been stated not to negatively influence the performance of implants; from 0.9 to 2.2 [21] or 0.6 to 2.36 [20]. From the SiG, only 2/88 implants slightly exceeded this second previously published range and 5/88 the first one. The differences between SiG and CG in the crown-to implant in the present study were lower than in other studies [20, 21] probably because the length of the CG implants was lower.

The use of transepithelial abutments in all the implants probably contributed to achieve a low number of technical complications in both groups and helped to dissipate implant loads thus positively influencing in the biological performance too [22, 49, 50]. This total homogeneity in the type of prosthetic restoration and in the type of implant surface together with a well-balanced sampling in relation to patients´ sex and age, number of smokers, bone type, insertion torque or type of antagonist, are relevant strengths of the present study. The unicentric design of the study ensured that all implants were placed and prosthetically rehabilitated by the same team, following the same protocol. This could have helped to reduce bias but at the same time the lack of data from other centers could be at the same time considered a limitation of the study. Other limitations of the study were the limited follow-up time or the reduced ability to control confounding factors of the retrospective designs. New long-term prospective studies are recommended to confirm these results.

## Conclusions

Within the limitations of this study, short (≤ 6.5 mm) and standard (≥ 7.5 mm) implants supporting a single-crown restoration over a transepithelial (intermediate abutment) in the posterior maxilla/mandible show similar clinical performance (survival, MBL, prosthetic complications).

## Data Availability

The data sets used and/or analysed during the current study are available from the corresponding author on reasonable request.

## References

[1] Araújo MG, Silva CO, Misawa M, Sukekava F (2000). Alveolar socket healing: what can we learn?. Periodontol.

[2] Jung RE, Ioannidis A, Hämmerle CHF, Thoma DS (2000). Alveolar ridge preservation in the esthetic zone. Periodontol.

[3] Nevins M, Camelo M, De Paoli S, Friedland B, Schenk RK, Parma-Benfenati S, Simion M, Tinti C, Wagenberg B (2006). A study of the fate of the buccal wall of extraction sockets of teeth with prominent roots. Int J Periodontics Restorative Dent.

[4] Tan WL, Wong TL, Wong MC, Lang NP (2012). A systematic review of post-extractional alveolar hard and soft tissue dimensional changes in humans. Clin Oral Implants Res.

[5] Atwood DA (1971). Reduction of residual ridges: a major oral disease entity. J Prosthet Dent.

[6] Buser D, Martin W, Belser UC (2004). Optimizing esthetics for implant restorations in the anterior maxilla: anatomic and surgical considerations. Int J Oral Maxillofac Implants.

[7] Tolentino da Rosa de Souza P, Binhame Albini Martini M, Reis Azevedo-Alanis L. Do short implants have similar survival rates compared to standard implants in posterior single crown? A systematic review and meta-analysis. Clin Implant Dent Relat Res. 2018;20:890–901. 10.1111/cid.12634 30051949

[8] Esposito M, Felice P, Worthington HV (2014). Interventions for replacing missing teeth: augmentation procedures of the maxillary sinus. Cochrane Database Syst Rev.

[9] Jerjes W, Hopper C (2018). Surgical experience, workload and learning curve vs postoperative outcome. Eur J Oral Implantol.

[10] Sakkas A, Schramm A, Winter K, Wilde F (2018). Risk factors for post-operative complications after procedures for autologous bone augmentation from different donor sites. J Craniomaxillofac Surg.

[11] Urban IA, Montero E, Monje A, Sanz-Sánchez I (2019). Effectiveness of vertical ridge augmentation interventions: A systematic review and meta-analysis. J Clin Periodontol.

[12] Pjetursson BE, Tan WC, Zwahlen M, Lang NP (2008). A systematic review of the success of sinus floor elevation and survival of implants inserted in combination with sinus floor elevation. J Clin Periodontol.

[13] Anitua E, Alkhraist MH, Piñas L, Begoña L, Orive G (2014). Implant survival and crestal bone loss around extra-short implants supporting a fixed denture: the effect of crown height space, crown-to-implant ratio, and offset placement of the prosthesis. Int J Oral Maxillofac Implants.

[14] Anitua E, Flores J, Flores C, Alkhraisat MH (2016). Long-term Outcomes of Immediate Loading of Short Implants: A Controlled Retrospective Cohort Study. Int J Oral Maxillofac Implants.

[15] Araki H, Nakano T, Ono S, Yatani H (2020). Three-dimensional finite element analysis of extra short implants focusing on implant designs and materials. Int J Implant Dent.

[16] do Vale Souza JP, Tavares Piacenza L, Penitente PA, Bueno Carlini Bittencourt AB, Dos Santos DM, Coelho Goiato M. Success rate of short unitary implants installed in atrophic mandible: Integrative Review. Clin Ter. 2022;173:180–183. 10.7417/CT.2022.2413 35385042

[17] Anitua E, Alkhraisat MH, Orive G (2013). Novel technique for the treatment of the severely atrophied posterior mandible. Int J Oral Maxillofac Implants.

[18] Lorenz J, Blume M, Korzinskas T, Ghanaati S, Sader RA (2019). Short implants in the posterior maxilla to avoid sinus augmentation procedure: 5-year results from a retrospective cohort study. Int J Implant Dent.

[19] Anitua E, Alkhraisat MH (2019). Single-unit short dental implants. Would they survive a long period of service?. Br J Oral Maxillofac Surg..

[20] Garaicoa-Pazmiño C, Suárez-López del Amo F, Monje A, Catena A, Ortega-Oller I, Galindo-Moreno P, Wang HL. Influence of crown/implant ratio on marginal bone loss: a systematic review. J Periodontol. 2014;85:1214–21. 10.1902/jop.2014.130615 24444399

[21] Hämmerle CHF, Cordaro L, Alccayhuaman KAA, Botticelli D, Esposito M, Colomina LE, Gil A, Gulje FL, Ioannidis A, Meijer H, Papageorgiou S, Raghoebar G, Romeo E, Renouard F, Storelli S, Torsello F, Wachtel H (2018). Biomechanical aspects: Summary and consensus statements of group 4. The 5th EAO Consensus Conference 2018. Clin Oral Implants Res.

[22] Anitua E, Larrazabal Saez de Ibarra N, Morales Martín I, Saracho Rotaeche L. Influence of dental implant diameter and bone quality on the biomechanics of single-crown restoration. A finite element analysis. Dent J (Basel). 2021;9:103. 10.3390/dj9090103 PMC8464909 34562977

[23] Anitua E, Alkhraisat MH (2019). Clinical performance of short dental implants supporting single crown restoration in the molar-premolar region: cement versus screw retention. Int J Oral Maxillofac Implants.

[24] Anitua E, Alkhraisat MH (2019). 15-year follow-up of short dental implants placed in the partially edentulous patient: Mandible Vs maxilla. Ann Anat.

[25] Papaspyridakos P, De Souza A, Vazouras K, Gholami H, Pagni S, Weber HP (2018). Survival rates of short dental implants (≤6 mm) compared with implants longer than 6 mm in posterior jaw areas: a meta-analysis. Clin Oral Implants Res.

[26] Mezzomo LA, Miller R, Triches D, Alonso F, Shinkai RS (2014). Meta-analysis of single crowns supported by short (<10 mm) implants in the posterior region. J Clin Periodontol.

[27] Badaró MM, Mendoza Marin DO, Pauletto P, Simek Vega Gonçalves TM, Porporatti AL, De Luca Canto G. Failures in Single Extra-Short Implants (≤ 6 mm): A Systematic Review and Meta-analysis. Int J Oral Maxillofac Implants. 2021;36:669–689. 10.11607/jomi.8689 34411206

[28] Vetromilla BM, Mazzetti T, Pereira-Cenci T (2021). Short versus standard implants associated with sinus floor elevation: An umbrella review of meta-analyses of multiple outcomes. J Prosthet Dent.

[29] Lin ZZ, Jiao YQ, Ye ZY, Wang GG, Ding X (2021). The survival rate of transcrestal sinus floor elevation combined with short implants: a systematic review and meta-analysis of observational studies. Int J Implant Dent.

[30] Torres-Alemany A, Fernández-Estevan L, Agustín-Panadero R, Montiel-Company JM, Labaig-Rueda C, Mañes-Ferrer JF (2020). Clinical behavior of short dental implants: systematic review and meta-analysis. J Clin Med.

[31] Lemos CA, Ferro-Alves ML, Okamoto R, Mendonça MR, Pellizzer EP (2016). Short dental implants versus standard dental implants placed in the posterior jaws: a systematic review and meta-analysis. J Dent.

[32] de N Dias FJ, Pecorari VGA, Martins CB, Del Fabbro M, Casati MZ. Short implants versus bone augmentation in combination with standard-length implants in posterior atrophic partially edentulous mandibles: systematic review and meta-analysis with the Bayesian approach. Int J Oral Maxillofac Surg. 2019;48:90–96. 10.1016/j.ijom.2018.05.009 29843950

[33] Nisand D, Picard N, Rocchietta I (2015). Short implants compared to implants in vertically augmented bone: a systematic review. Clin Oral Implants Res.

[34] Kotsovilis S, Fourmousis I, Karoussis IK, Bamia C (2009). A systematic review and meta-analysis on the effect of implant length on the survival of rough-surface dental implants. J Periodontol.

[35] Annibali S, Cristalli MP, Dell'Aquila D, Bignozzi I, La Monaca G, Pilloni A (2012). Short dental implants: a systematic review. J Dent Res.

[36] Telleman G, Raghoebar GM, Vissink A, den Hartog L, Huddleston Slater JJ, Meijer HJ (2011). A systematic review of the prognosis of short (<10 mm) dental implants placed in the partially edentulous patient. J Clin Periodontol.

[37] Wang Y, Jiang J, Guan Y, Si M, He F (2021). Retrospective study of short versus standard posterior implants and analysis of implant failure risk factors. Int J Oral Maxillofac Implants.

[38] Al-Johany SS. Survival Rates of Short Dental Implants (≤ 6.5 mm) Placed in Posterior Edentulous Ridges and Factors Affecting their Survival after a 12-Month Follow-up Period: A Systematic Review. Int J Oral Maxillofac Implants. 2019;34:605–621. 10.11607/jomi.7187 30703180

[39] Carosi P, Lorenzi C, Lio F, Laureti M, Ferrigno N, Arcuri C (2021). Short implants (≤6mm) as an alternative treatment option to maxillary sinus lift. Int J Oral Maxillofac Surg.

[40] Nielsen HB, Schou S, Bruun NH, Starch-Jensen T (2021). Single-crown restorations supported by short implants (6 mm) compared with standard-length implants (13 mm) in conjunction with maxillary sinus floor augmentation: a randomized, controlled clinical trial. Int J Implant Dent.

[41] Xu X, Hu B, Xu Y, Liu Q, Ding H, Xu L (2020). Short versus standard implants for single-crown restorations in the posterior region: A systematic review and meta-analysis. J Prosthet Dent.

[42] Anitua E, Alkhraisat MH, Piñas L, Orive G (2015). Efficacy of biologically guided implant site preparation to obtain adequate primary implant stability. Ann Anat.

[43] Misch CE, Perel ML, Wang HL (2008). Implant success, survival, and failure: the International Congress of Oral Implantologists (ICOI) Pisa Consensus Conference. Implant Dent.

[44] Rameh S, Menhall A, Younes R (2020). Key factors influencing short implant success. Oral Maxillofac Surg.

[45] Rossi F, Botticelli D, Cesaretti G, De Santis E, Storelli S, Lang NP (2016). Use of short implants (6 mm) in a single-tooth replacement: a 5-year follow-up prospective randomized controlled multicenter clinical study. Clin Oral Implants Res.

[46] Anitua E, Tapia R, Luzuriaga F, Orive G (2010). Influence of implant length, diameter, and geometry on stress distribution: a finite element analysis. Int J Periodontics Restorative Dent.

[47] e Souza Batista VE, Verri FR, Lemos CA, Cruz RS, Noritomi PY, Pellizzer EP. A 3D finite element analysis of bone tissue in 3-unit implant-supported prostheses: effect of splinting factor and implant length and diameter. Eur J Prosthodont Restor Dent. 2021;29:76–83. 10.1922/EJPRD_2098deSouzaBatista08 33146474

[48] Ravidà A, Galli M, Bianchi M (2021). Clinical outcomes of short implants (≤ 6 mm) placed between two adjacent teeth/implants or in the most distal position: a systematic review and meta-analysis. Int J Oral Implantol (Berl).

[49] Anitua E, Piñas L, Orive G (2015). Retrospective study of short and extra-short implants placed in posterior regions: influence of crown-to-implant ratio on marginal bone loss. Clin Implant Dent Relat Res.

[50] Hernández-Marcos G, Hernández-Herrera M, Anitua E (2018). Marginal bone loss around short dental implants restored at implant level and with transmucosal abutment: a retrospective study. Int J Oral Maxillofac Implants.

